# Collaborating with patient and public members in developing the COVID - Curated and open analysis and research platform (CO-CONNECT).

**DOI:** 10.23889/ijpds.v7i3.1781

**Published:** 2022-08-25

**Authors:** Tracy Jackson, Jillian Beggs, Antony Chuter, Marialuisa Cursino, Aziz Sheikh

**Affiliations:** 1 University of Edinburgh; 2 University of Nottingham

## Objectives

We aimed to support the work of CO-CONNECT by meaningfully involving patient and public members across all project work packages. In addition, we aimed to ensure that the team members and outputs are connected to public perspectives and that public voices are adequately represented and embedded throughout CO-CONNECT.

## Approach

We have two public members on our leadership team who co-lead our Patient and Public Involvement and Engagement (PPIE) work stream with support from academics. They convened a “Public User Group” (PUG) of five public members from across the United Kingdom who regularly contribute to all aspects of CO-CONNECT. Our PPIE work was co-produced with our public members and a PPIE strategy was developed to ensure meaningful involvement throughout CO-CONNECT. At the beginning of the project, we developed an information pack for our public members to provide insight into CO-CONNECT and the importance of their role.

## Results

To ensure complete transparency with the public, our PUG members attend and actively contribute to all team meetings within CO-CONNECT. This provides opportunities for public voices to be heard and acted upon in relation to questions about the use of, and access to, healthcare data in healthcare research. PUG members have contributed to the development of the CO-CONNECT website including providing information for biographies to increase public awareness of the involvement of public members in CO-CONNECT. They have written blogs and been interviewed for newsletter articles on the important of public involvement in research. Together we have created videos discussing their experience of being involved with CO-CONNECT and created a set of “Frequently Asked Questions” to provide more information about CO-CONNECT for the public-facing website.

## Conclusion

The PPIE work within CO-CONNECT has created an innovative approach to ensuring public voices are heard and acted upon within data linkage networks. This model has the potential to be used in future projects to ensure inclusive and meaningful involvement of patient and public members in healthcare research.

